# Long-term ω-3 fatty acid supplementation induces anti-stress effects and improves learning in rats

**DOI:** 10.1186/1744-9081-9-25

**Published:** 2013-06-14

**Authors:** Miguel Á Pérez, Gonzalo Terreros, Alexies Dagnino-Subiabre

**Affiliations:** 1 Laboratory of Behavioral Neurobiology, Centro de Neurobiología y Plasticidad Cerebral, Departamento de Fisiología, Facultad de Ciencias, Universidad de Valparaíso, Gran Bretaña 1111, Playa Ancha, Valparaíso, Chile; 2 Graduate Program in Biology and Ecology Applied, Universidad Católica del Norte, Coquimbo, Chile

**Keywords:** Stress, ω-3 polyunsaturated fatty acid, Anxiety, Learning

## Abstract

Chronic stress leads to secretion of the adrenal steroid hormone corticosterone, inducing hippocampal atrophy and dendritic hypertrophy in the rat amygdala. Both alterations have been correlated with memory impairment and increased anxiety. Supplementation with ω-3 fatty acids improves memory and learning in rats. The aim of this study was to evaluate the effects of ω-3 supplementation on learning and major biological and behavioral stress markers. Male Sprague–Dawley rats were randomly assigned to three experimental groups: 1) Control, 2) Vehicle, animals supplemented with water, and 3) ω-3, rats supplemented with ω-3 (100 mg of DHA+25 mg of EPA). Each experimental group was divided into two subgroups: one of which was not subjected to stress while the other was subjected to a restraint stress paradigm. Afterwards, learning was analyzed by avoidance conditioning. As well, plasma corticosterone levels and anxiety were evaluated as stress markers, respectively by ELISA and the plus-maze test. Restraint stress impaired learning and increased both corticosterone levels and the number of entries into the open-arm (elevated plus-maze). These alterations were prevented by ω-3 supplementation. Thus, our results demonstrate that ω-3 supplementation had two beneficial effects on the stressed rats, a strong anti-stress effect and improved learning.

## Introduction

Stress is a complex biological reaction common to all living organisms that allows them to adapt to environmental pressure (i.e., stressors) [1,2]. Stress responses are mainly mediated by the activation of the hypothalamic-pituitary-adrenal (HPA) axis, leading to secretion of glucocorticoids from the adrenal gland; glucocorticoids are bound to glucocorticoid receptors in peripheral tissues and the brain to regulate stress responses [3-5]. Stressors increase the release of corticotrophin releasing factor (CRF) from the hypothamus, inducing adrenocorticotropic hormone release from the anterior pituitary, which in turn stimulates the secretion of corticosterone from the adrenal cortex [6]. Corticosterone is bound to glucocorticoid receptors (GRs) in peripheral tissues and the brain [3,5,7]. The hippocampus, amygdala and medial prefrontal cortex have high concentrations of GRs [8-10]. Chronic glucocorticoid treatment induces dendritic atrophy in the hippocampus [11-13] and medial prefrontal cortex [13], while acute corticosterone treatment induces dendritic hypertrophy in the basolateral amygdaloid nucleus and enhances anxiety [14-16].

Chronic stress and corticosterone treatment affect the dendritic morphology of limbic areas of the rat brain, such as the hippocampus, amygdaloid complex, and prefrontal cortex [17-19]. These alterations increase anxiety, and impair both memory and spatial learning [20-22]. Anxiety is an adaptive reaction induced when an animal is confronted with potential demands and dangers. Indeed, anxiety has a key biological-adaptive role, which is highly conserved during evolution [23]. Excessive or pathological levels of anxiety induce maladaptive responses [23]. In humans, chronic stress or psychosocial stress also produces hippocampal volume atrophy [24] and functional changes in the prefrontal cortex [25].

Learning in animal models involves associating an auditory cue (conditioned stimulus, CS) with an aversive unconditioned stimulus (US). Once learned, the CS will by itself elicit a conditioned response. For instance, freezing is a conditioned response to fear in two-way signaled active avoidance conditioning (2-AA). Rats are trained in a shuttle box to avoid a foot shock signaled by an auditory cue [26]. Chronic restraint stress impaired the learning in the 2-AA and the neuronal morphology of the auditory system [27,28].

There is abundant evidence that chronic stress and/or diet affects the brain physiology in animal models as well as in humans. However, the interaction between stress and diet is poorly understood [29]. Docosahexaenoic acid (22:6 ω-3; DHA) is a predominant dietary ω-3 polyunsaturated fatty acid (PUFAs) that improves learning by animals in the 2-AA paradigm [30]. Conversely, rats subjected to maternal separation and a ω-3 PUFA-deficient diet are more anxious and fearful than control animals [31]. Long-term EPA and DHA supplementation decreases anger and anxiety in animal models of drug addiction [32,33]. In humans, PUFAs have positive effects on the pathophysiology of a wide range of stress-related disorders [32,34-37]. Patients suffering major depression, a stress-related disorder, have shown reductions in the plasma levels of ω-3 PUFAs, without any change in fatty acid ω-6 levels [38-41].

These findings suggest that long-term ω-3 supplementation of the diet of post-weaning animals has two effects on chronically stressed rats: preventing the stress-induced learning impairment in a 2-AA test and decreasing plasma corticosterone levels and anxiety. The objective of this study was to analyze the effects of a DHA and EPA mix on learning, corticosterone levels and anxiety of Sprague–Dawley rats subjected to a chronic restraint-stress paradigm.

## Materials and methods

### Animals

Male *Sprague–Dawley* rats (80–100 g, 21 days old at the start of the experiment) were housed in groups of three animals per cage, under a 12/12 light/dark cycle (lights on at 8:00 am). They were maintained in a temperature and humidity controlled room (20 ± 1°C, 55%) and weighed every day on a digital scale (Model WLC2/A1, Radwag, Poland). All procedures relating to animal experimentation were in strict accordance with animal care standards outlined in the National Institute of Health (USA) guidelines and approved by the Institutional Animal Ethics Committee of the Universidad de Valparaíso and Universidad Católica del Norte. Efforts were made to minimize the number of animals used and their suffering.

### Experimental design

**Figure 1:**
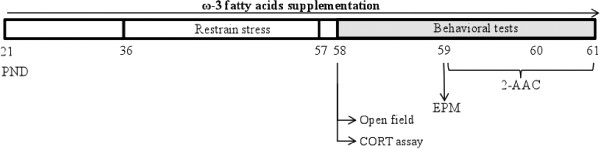
**Schematic drawing of the experimental design.** Experimental time line (not to scale) from Post Natal Day (PND) 21 to 61 (ω-3 fatty acids supplementation). Restraint stress was applied from PND 36 to 57; EPM: elevated plus maze test; 2-AA: Active avoidance conditioning test.

Figure 1 shows a schematic drawing of the experimental design used in this study. Rats were maintained with *ad libitum* access to food (rat chow, Champion®, Santiago, Chile) and water during all experiments. Each rat ate between 15–30 g of rat chow per day; 0.34% of 1 g of rat chow was ω-3 fatty acids. Rats were randomly assigned into three experimental groups for supplementation: Control (*n* = 60), animals did not receive supplementation; Vehicle (*n* = 60), rats were supplemented daily with 2.5 ml of water by oral administration; and ω-3-supplemented animals (*n* = 60), which were supplemented daily with 2.5 ml of a mix of 100 mg of DHA and 25 mg of EPA per kg animal weight by oral administration (Knop Laboratories S.A. Santiago, Chile). Supplementation was applied once per day; the rat was picked up from its home cage and gently held in the hand of the experimenter for the oral administration of vehicle or ω-3. The procedure took approximately one minute. Animals from the vehicle and ω-3 groups received water or ω-3 fatty acids respectively between postnatal days (PND) 21 and 61 (Figure 1). Each experimental group was divided into two subgroups: one of which was not subjected to any type of stress [control + unstressed (U), C-U, *n* = 30; vehicle + unstressed, V-U, *n* = 30; ω-3 + unstressed, ω-3-U, *n* = 30], while the other one was subjected to a restraint-stress protocol (control + stress, C-S, *n* = 30; vehicle + stress, V-S, *n* = 30; ω-3 + stress, ω-3-S, *n* = 30). Stressed and unstressed animals were littermates and after weaning were housed in separate rooms. Unstressed rats were never exposed to stressed and the restraint stress was applied in different room. Table 1 shows the number of animals used in each experiment.

**Table 1:** Number of rats used in each experiment

	**Unstressed rats**			**Stressed rats**			
**Experiment**	**Control**	**Vehicle**	**ω-3**	**Control**	**Vehicle**	**ω-3**	**Total**
Locomotor activities and Anxiety	*n* = 9	*n* = 9	*n* = 9	*n* = 9	*n* = 9	*n* = 9	*n* = 54
CORT levels in unstimulated rats	*n* = 6	*n* = 6	*n* = 6	*n* = 6	*n* = 6	*n* = 6	*n* = 36
CORT levels after acute swim	*n* = 6	*n* = 6	*n* = 6	*n* = 6	*n* = 6	*n* = 6	*n* = 36
Learning (active avoidance conditioning)	*n* = 9	*n* = 9	*n* = 9	*n* = 9	*n* = 9	*n* = 9	*n* = 54
**Total**	30	30	30	30	30	30	***n *= 180**

### Handling procedure and restraint stress

Rats were removed every day by hand and transferred to another cage on the pan of a balance to be weighed. Different investigators did this procedure from those applying the restraint stress. All rats in every group were handled with the same procedures. Animals were placed in acrylic restrainers (6 cm wide × 12 cm long and then 6 cm wide × 20 cm long as the rats grew) in their home cages. They were subject to restriction for 6 h every day, beginning at 10 am, from the 36 to 57 PND (21 days of restraint stress). Restrainers were perforated at each end to allow ventilation and avoid overheating the animals. During the stress protocol, animals could breathe without difficulty and urinate and defecate without being in constant contact with their wastes. The following additional parameters were measured to monitor the overall effects of the stress protocol: percentage gain in body weight, plasma corticosterone levels, and anxiety (see below).

### Behavioral procedures

On day one and day two after the end of the stress protocol rats were analyzed individually in the open field and the EPM respectively. A separate set of rats was used for this experiment (C-U, *n* = 9; C-S, *n* = 9; V-U, *n* = 9; V-S, *n* = 9; ω-3-U, *n* = 9; ω-3-S, *n* = 9). Behavioral tests were carried out from 10 am to 2 pm in the test room. The activity of each rat was recorded by Internet Protocol (IP) cameras (VIVOTEK, Sunnyvale CA, USA) fixed above the behavioral apparatus and connected to a computer in another room. Videos were acquired by Nuuo software (Nuuo, Taipei, Taiwan) and analyzed with ANY-maze video tracking system (Stoelting Co., IL, USA). Mazes were wiped and cleaned with 5% ethanol solution after each trial. In all experiments, animals from control and stress groups were evaluated at the same time.

### Open field test

Behavior tests were conducted in a soundproof and temperature-controlled (21 ± 1°C) room. Each rat was placed in the center of a black Plexiglass cage (70 × 70 × 40 cm) for 5 min. The background noise level in the open field was 40 dB (Precision sound level meter, Model # 1100, Quest Technologies, Oconomowoc, WI) and the arena was illuminated by 300 ± 20 lux (measured by a digital lux meter, Model # LX-1010B, Weafo Instrument Co., Shanghai, China). The total distance travelled and average speed were determined from the video recordings and analyzed with the ANY-maze video tracking system (Stoelting Co., IL, USA).

### Elevated Plus-Maze (EPM)

Twenty-four hours after the open field test, we measured anxiety levels using an EPM test. Each rat was placed individually in an EPM, consisting of two open arms (60 × 15 cm each), two closed arms (60 × 15 × 20 cm each) and a central platform (15 × 15 cm) arranged so that the two arms of each type were opposite to each other. The maze was elevated 100 cm above the floor. The illumination was 300 ± 10 luxs in the open arms and 210 ± 10 luxs in the closed arms. At the beginning of each trial, animals were placed at the center of the maze, facing an open arm. During a 5-min test period we recorded the frequency of entries to the open and closed arms. The percentage of entries and the ratio of open to total arm entries (open/total×100) were used as measures of the anxiety level. Total arm entries were taken as an indicator of general locomotor activity. Entry into an arm was defined as having occurred when the animal placed all four limbs onto the arm.

### Active avoidance conditioning (2-AA)

#### Apparatus

Rats were trained in a shuttle box (50 × 25 × 25 cm3) that was divided into two identical stainless steel modular testing units by a black Plexiglas divider. The divider had a narrow passage (8 cm) opened between the sections. The grid floor had 38 stainless steel bars arranged parallel to the dividers (Panlab Instruments, Barcelona, Spain). A 3-kHz 80-dB tone was presented to subjects as a conditioned stimulus (CS) signaling the upcoming unconditioned stimulus (US) in the form of a foot shock (0.5 mA) delivered by a shocker (LE 100–26, Panlab S.L., Barcelona, Spain). The CS was delivered simultaneously by speakers located on opposite walls of the chamber (20 cm high). Both the CS and US stimuli were regulated by Shutavoid software (Panlab S.L., Barcelona, Spain). The conditioning chamber was placed in a sound-attenuating box. The inside of the box was dimly illuminated with a 0.5-W LED bulb.

### Behavioral training

A separate set of rats was used for this experiment (C-U, *n* = 9; C-S, *n* = 9; V-U, *n* = 9; V-S, *n* = 9; ω-3-U, *n* = 9; ω-3-S, *n* = 9). The rats were placed in the shuttle box and trained individually. During the training sessions the rats were subjected to a 5-min stimulus-free acclimation period. On day 1, all rats were first exposed to a 5-min acclimation period, followed by the habituation trials (habituation) where rats received a CS tone for 20 sec, with an average inter-trial interval (ITI) of 30 s without presenting the US. Rats were then returned to their home cages. On day 2 (conditioning day 1), after an acclimation period, rats received 100 signaled avoidance trials with an average ITI of 30 sec. Each trial consisted of 20 sec of CS, the last 10 sec of which coincided with a 10-sec US. Shuttling action by the rat cut the tone immediately and prevented the foot shock. If there was no shuttling during the 20-sec tone, the foot shock was applied until the rat shuttled (escape response) or the shock continued for a maximum of 10 sec. Rats were then returned to their home cages for 24 h. On day 3 (conditioning day 2), rats were returned to the chamber and received 50 signaled avoidance trials with an average ITI of 30 sec, the last 10 sec of which overlapped with a 0.5-mA foot shock (maximum shock duration of 10 s) until the animal escaped to the opposite chamber.

### Behavioral measurement

All animal movements were recorded by IP cameras mounted inside the sound-attenuating box. Conditioned avoidance response (CR) was defined as the rat crossing to the opposite chamber within the first 10 s after the tone started. One hundred and fifty training trials were applied to all animals on days 2 and 3, which were divided into fifteen blocks of 10 trials each. The number of CRs was measured in each block of trials and the percentage of CRs (% CR) was calculated [(number of CR/10 training trials)×100]. All data were measured by Shutavoid software (Panlab S.L., Barcelona, Spain).

### Plasma corticosterone measurement by ELISA

This experiment analyzed whether restraint stress affects the stress levels of the rats one day after the stress had ended. The most conventional method to determine if animals are stressed is to measure the plasma levels of the stress hormone corticosterone. Stressed animals show an increase in HPA axis activity and plasma corticosterone levels compared to controls after exposure to an uncontrollable stressor, leading to maladaptive responses [42]. In this way, acute swim stress in a water maze increases plasma corticosterone levels of *Sprague–Dawley* rats [43]. Therefore, we measured the plasma corticosterone levels of the rats one day after of the last restraint session, when behavioral experiments were initially conducted.

Animals were subjected to a new stressor (swimming in a water maze) and corticosterone plasma levels were quantified before and after water maze exposure.

A separate set of animals was used to measure the concentration of corticosterone in plasma to avoid the stressfulness of blood collection being a contaminating factor in the behavioral experiments. One set of rats (C-U, *n* = 6, C-S, *n* = 6, V-U, *n* = 6, V-S, *n* = 6, ω-3-U, *n* = 6, ω-3-S, *n* = 6) was given a 60 s probe trial in a water maze at 11 am after which the animals were transferred to a heated holding cage for 10 minutes. Afterward, the animals were taken to a separate room (time used approximately 10 s) and quickly anesthetized with isoflurane (time used approximately 5 s) and immediately sacrificed via decapitation under deep anesthesia for blood collection. Animals were not exposed to other decapitated animals before being anesthetized. Another set of rats (C-U, *n* = 6, C-S, *n* = 6, V-U, *n* = 6, V-S, *n* = 6, ω-3-U, *n* = 6, ω-3-S, *n* = 6) was not disturbed and was sacrificed at 11:11 am under deep anesthesia. The Morris water maze consisted of a blue circular tank (183 cm diameter) in a room that was rich with spatial cues. The tank contained non-toxic colored water at 19°C (black non-toxic tempura paint).

Blood (1 ml) was collected in heparinized tubes, centrifuged at 3,000 rpm (Model # MiniSpin Plus; Eppendorf AG, Hamburg, Germany) for 20 min to obtain plasma, which was then stored at −70°C. Total corticosterone was determined by an Enzyme Immunoassay kit (CorticosteroneBioAssay™, Catalog. # C7903-30) purchased from US Biological (Swampscott, MA). Optical density values were measured at 450 nm using a micro-plate reader (Model # Anthos 2010 Microplate Reader, Biochrom Ltd, UK). Samples were diluted 1:10 and processed in duplicates. Averaged final values were represented as μg/dL.

### Statistical analysis

#### Open field test and percentage of body weight gain

Time, total distance travelled, and average speeds were analyzed with the Student’s *t*-test. Percentage of body weight gain was analyzed using a two-way repeated-measures ANOVA [groups (control, stress) × days (1, 7, 14, 21)] followed by Bonferroni *post-hoc* comparison tests. A two-way ANOVA compared groups for anxiety levels in the open field test. The dependent variable for anxiety was the time spent in the center of the open field and the independent variables were restraint stress (unstressed and stressed) and the diet (control, vehicle and ω-3).

### Anxiety and corticosterone levels

A two-way ANOVA compared groups for anxiety levels in the plus-maze. The dependent variable for anxiety was the percentage of open-arm entries and the independent variables were restraint stress (unstressed and stressed) and diet (control, vehicle and ω-3). The corticosterone levels were analyzed by a 3 × 2 factorial ANOVA.

### Active avoidance

The CR percentage was analyzed with a two-way repeated-measures ANOVA [groups (control, stress)×trials (habituation, conditioning day 1, conditioning day 2)] followed by a Bonferroni *post-hoc* comparisons test. Data from the % CR were transformed to arcsine [(arcsine of square root of (% CR/100)] to satisfy requirements of the ANOVA model and then the slopes were analyzed by regression analysis.

## Results

### Body weight gain and locomotor activity

**Figure 2:**
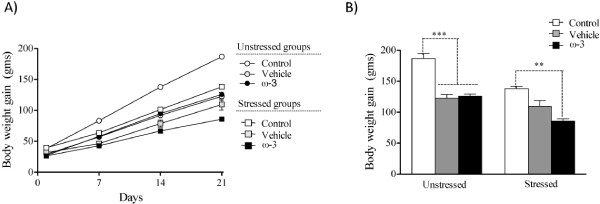
**Effect of chronic restraint stress on body weight gain.** Rats subjected to restraint stress failed to gain weight (**A** and **B**). Differences in body weight gain were observed from day 7 between stressed and unstressed rats from both control and ω-3 groups, and from day 14 for animals from the vehicle groups (**A**). In contrast, unstressed rats gained weight throughout the study. ω-3: ω-3 supplementation. Data are represented as means ± SEM. An asterisk (*) indicates significant differences.

The two-way repeated-measures ANOVA showed that chronic restraint stress significantly reduced body weight gain beginning on PND 21 (*F*_(1,48)_ = 35.61, *p* < 0.0001) (Figure 2A). There was a main effect of diet and interaction on body weight gain, where the post-hoc test showed that rats subjected to restraint stress from both control and ω-3 diet groups had significantly decreased body weight gain compared to the respective unstressed controls (*F*_(1,48)_ = 1559, *p* < 0.0001). Conversely, body weight gain of rats from the vehicle (diet) group subjected to restraint stress was not affected compared to unstressed rats from the vehicle group (Figure 2A). Figure 2B shows body weight gain at PND 21 (same data as in Figure 2A). There was a main effect of both stress and diet where the body weight gain of rats subjected to the stress protocol was significantly lower (*F*_(2,48)_ = 35.612, *p* < 0.0001), while rats subjected to stress that received supplements (vehicle and ω-3 diet) had significantly lower body weight gain than rats on the control diet (*F*_(2,48)_ = 48.701, *p* < 0.0001). There was a significant effect of the diet-stress interaction on body weight gain (*F*_(2,48)_ = 3.46, *p* < 0.05). The post-hoc test showed that rats of the control and ω-3 groups that were subjected to restraint stress had significantly lower body weight gain than unstressed rats (*F*_(2,48)_ = 3.46, *p* < 0.001). Conversely, restraint stress did not affect the body weight gain of animals in the vehicle group (*F*_(2,48)_= 3.46, *p* = 0.849).

Restraint stress did not affect locomotor activity levels (total distance traveled and average speed) in any experimental group (Table 2). Restraint stress significantly reduced time spent in the center of open field among control and vehicle groups rats (*F*_(1,48)_ = 9.06, *p* < 0.01), while stress did not affect ω-3 group rats. Animals of the vehicle group spent significantly more time in the center of open field than control group rats (*F*_(2,48)_ = 4.93, *p* < 0.01). A two-way ANOVA did not show an interaction between restraint stress and diet (Table 2).

**Table 2:** Locomotor activity of the animals

**Groups**	**Subjects**	**Total distance traveled (m)**	**Average speed (m)**	**Time spent in center of open field (sec)**
***Unstressed groups***				
Control	9	23.1 ± 3.1	0.07 ± 0.01	19.0 ± 3.04
Vehicle	9	20.3 ± 1.8	0.07 ± 0.01	26.5 ± 2.88
ω-3	9	24.1 ± 3.4	0.08 ± 0.01	16.0 ± 1.53
***Stressed groups***				
Control	9	22.6 ± 3.6	0.07 ± 0.01	9.00 ± 2.22
Vehicle	9	18.4 ± 1.3	0.06 ± 0.004	16.8 ± 2.01
ω-3	9	21.2 ± 1.9	0.07 ± 0.01	16.3 ± 4.48

For each experiment shows the means value ± SEM.

### Effect of ω-3 fatty acids supplementation on anxiety and corticosterone plasma levels

**Figure 3:**
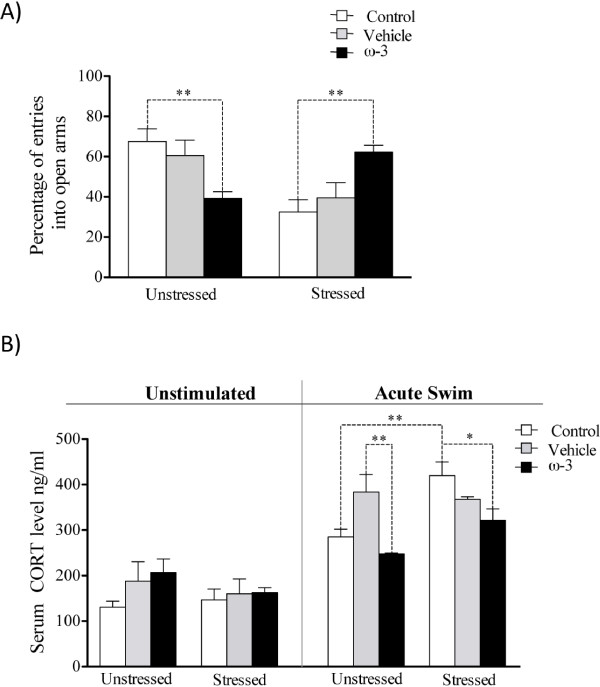
**Effects of ω-3 supplementation on stress markers.** (**A**) Restraint stress decreased the % of entries into the open arms of both control and vehicle group rats (*p* < 0.05). Supplementation with ω-3 increased the % of entries into the open arms among the stressed rats compared to unstressed animals (*p* < 0.05), producing an anxiolytic effect. (**B**) After acute swim, restraint stress significantly increased plasma corticosterone levels, but supplementation with ω-3 prevented this alteration. Error bars indicate the means ± SEM. An asterisk (*) indicates significant differences.

Restraint stress and vehicle treatment significantly decreased the percentage of open-arm entries (control group: stressed rats: 32.42 ± 6.24 entries, unstressed rats: 67.58 ± 6.24 entries, *p* = 0.002; vehicle group: stressed rats: 39.44 ± 7.63 entries, unstressed rats: 60.56 ± 7.64 entries; *p* > 0.150). This effect was prevented with ω-3 supplementation (stressed rats: ω-3 supplementation: 62.20 ± 3.42 entries, vehicle-treated rats: 39.44 ± 7.63 entries, control rats: 32.42 ± 6.24 entries, (*F*_(1,48)_ = 5.11, *p* = 0.03) (Figure 3A).

Figure 3B shows the level of circulating corticosterone in rats subjected to 60-s probe trials in the water maze and in animals that were not disturbed. There were no significant differences in the corticosterone levels between unstressed and stressed rats that were left undisturbed (Figure 3B, left side). Rats that were subjected to acute swim had significantly higher plasma corticosterone levels than rats that were not disturbed (*F*_(1,66)_ = 107.98, *p* < 0.001) (Figure 3B, right side). In the control group, rats that were subjected to restraint stress and swimming for 60 s in the water maze had higher corticosterone levels than rats that were unstressed (stressed = 420.0 ± 30.2 ng/ml, n = 6, unstressed = 285.1 ± 16.9 ng/ml, n = 6, p < 0.01) (Figure 3B). This effect was prevented by ω-3 supplementation (acute swimming; ω-3 group: stressed = 321.5 ± 25.3 ng/ml, n = 6, unstressed = 247.5 ± 2.5 ng/ml, n = 6, p > 0.05). After acute swimming, vehicle treatment significantly increased the plasma corticosterone levels in the unstressed rats compared to that of unstressed rats of control group (unstressed rats of vehicle group: 383.6 ± 38.5 ng/ml, n = 6, unstressed rats of control group: 285.1 ± 16.9 ng/ml, n = 6, p > 0.05).

### Effects of ω-3 fatty acids supplementation on active avoidance conditioning

**Figure 4:**
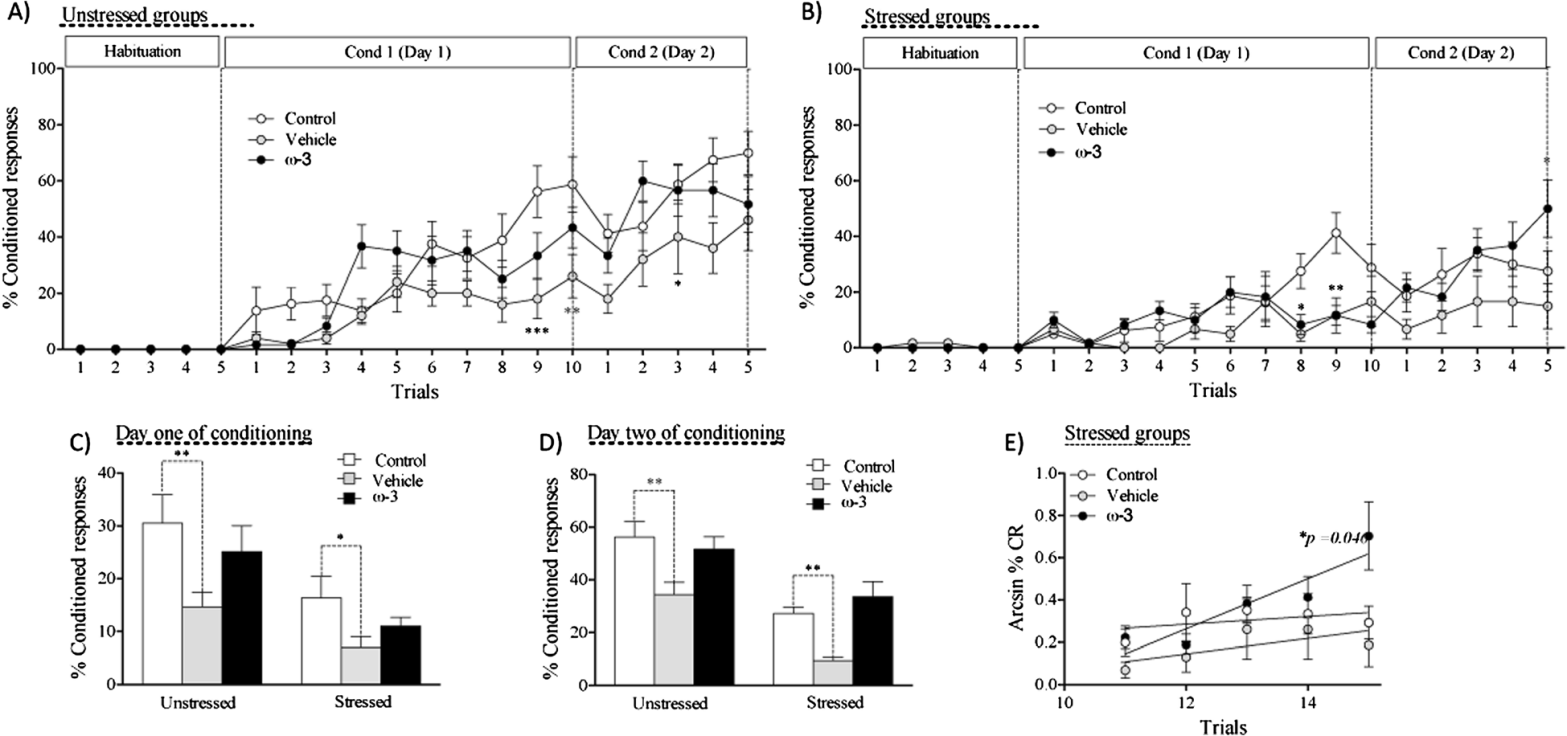
**Effects of ω-3 supplementation on learning. A** and **B**: Restraint stress decreased the percentage of conditioned responses but ω-3 prevented this effect. The values are the means ± SEM of 9 animals per group. Each point represents the percentages of conditioned avoidance responses (% CR) for the three stages of the test (habituation, conditioning one and two). **C**, **D**: Effect of ω-3 supplementation on learning during days 1 and 2 of conditioning, vehicle supplementation decreased learning, while ω-3 supplementation prevented this effect. **E**: Comparing the slopes of conditioned responses of rats subjected to restraint stress on day 2 of conditioning. The supplemented rats subjected tostress showed significant differences from the stressed rats in both the control and vehicle groups. ω-3: ω-3 supplementation. Data are represented as means ± SEM. An asterisk (*) indicates significant differences.

Figures 4A and B show the percentage of conditioned responses (% CR) during the avoidance conditioning to the tone under the three dietary regimes in unstressed and stressed conditions. The two-way repeated-measures ANOVA shows the main effects of the trials and interactions (trials* diet) on % CR of the unstressed rats (Figure 4A). There was no main effect of diet on % CR in rats of the unstressed group (*F*_(2,34)_ = 2.87, *p* = 0.07). Likewise, in the stressed groups, there was a main effect of trials and interaction (trials*diet) on % CR (Figure 4B). A 2 × 3 factorial ANOVA showed that chronic restraint stress significantly reduced the % CR on day 1 of conditioning (*F*_(1,48)_ = 15.417, *p* < 0.01) (Figure 4C). There was a main effect of diet on % CR (*F*_(2,48)_ = 5.779, *p* < 0.01), where the post-hoc test showed that the vehicle groups had significantly lower % CR compared to the control group. This effect was prevented by ω-3 supplementation (Figure 4C).

Figure 4D shows the % CR on day 2 of conditioning (Day 2). A 2 × 3 factorial ANOVA showed a main effect of chronic restraint stress on % CR (*F*_(1,48)_ = 46.949, *p* < 0.001), where the post-hoc test indicates that restraint stress significantly decreased the % CR compared to unstressed rats. There was a main effect of diet on % CR (*F*_(2,48)_ = 13.671, *p* < 0.001), where the post hoc test shows that the vehicle group had a significantly lower % CR than the control group. This effect was prevented by ω-3 supplementation (Figure 4D). Regression analysis to compare the slopes of stressed rats on day 2 of conditioning showed that animals from the ω-3 group had a steeper slope than rats of the other groups subjected to stress (*F*_(1,176)_ = 2.9, *p* =0.046) (Figure 4E).

## Discussion

In this study we analyzed the effects of ω-3 supplementation on anxiety, plasma corticosterone levels, and learning of chronically stressed rats. First, we investigated whether our stress protocol was effective in triggering stress responses. Stressed rats of all experimental groups had less body weight gain than unstressed control group rats (Figure 2A,B). This demonstrates that the stress protocol used was effective and that ω-3 supplementation did not prevent this effect (Figure 2A,B). Comparable results have been reported using similar stress paradigms and corticosterone administration [44-46]. Diets enriched with PUFAs, in particular the ω-3 family, decreased both the adipose tissue mass and plasma leptin levels in rats [47]. Leptin is released from adipocytes and regulates food intake and body weight by binding to leptin receptors to the hypothalamus [48,49]. Thus, it is probable that the ω-3 supplementation used in our experiments decreased the body weight of the rats compared to that of controls (Figure 2A,B).

### Effects of restraint stress and ω-3 fatty acid on anxiety and corticosterone plasma levels

Stressed control and vehicle groups rats had significantly lower percentages of entries into the open arms of the EPM and spent less time in the center of the open field test than unstressed rats (Figure 3A, Table 2). These behaviors were related to an anxiogenic effect induced by restraint stress and vehicle treatment (Figure 3A). Interestingly, supplementation with ω-3 had an opposite effect on the stressed rats. Indeed, ω-3 supplementation increased the number of entries into the open arm of the EPM, which was related to an anxiolytic effect (Figure 3A) that was not associated with locomotor impairments due to restraint stress and vehicle (Table 2).

Comparable results were obtained using another chronic restraint stress paradigm and ω-3 supplementation [50]. Anxiety is mainly regulated by the basolateral amygdala and the bed nucleus of the stria terminalis (BNST) [51-53]. In some chronic stress paradigms, such as chronic unpredictable stress or immobilization, enhanced anxiety has been correlated with dendritic hypertrophy in the basolateral amygdala and BNST [18,54,55]. It is possible that the chronic stress protocol and vehicle treatment in our study induced hyperactivation of the basolateral amygdala and/or BNST by plastic neuronal changes, which significantly increased anxiety, HPA axis activity, and plasma corticosterone levels (Figure 3A,B). Another brain area that modulates BNST neuronal excitability is the dorsal raphé nucleus (DRN) [56]. Axons of the serotoninergic neurons located in the DRN are sent to the BNST and serotonin is released to the synaptic space, which in turn inhibits the neuronal excitability in the BNST by activation of the 5-HT_1A_ and 5-HT_1B_ receptors [56,57]. Serotonin levels are reduced in the brain of stressed rats [58,59], and thereby the 5-HT_1A_ and 5-HT_1B_ receptors in the BNST are not activated. This alteration may contribute to increasing neuronal excitability in the BNST and anxiety in the stressed rats (Figure 3A).

On the other hand, ω-3 supplementation significantly decreased corticosterone levels in the unstressed and stressed rats (Figure 3A, right side). This suggests that ω-3 supplementation prevents hyperactivation of the HPA axis induced by chronic stress, decreasing the effects of corticosterone on dendritic morphology and neuronal activity of the basolateral amygdala and BNST. Supplementation with ω-3 increases serotonin levels in the brain of stressed rats [60], which in turn may reduce BNST neuronal excitability by activation of the 5-HT_1A_ and 5-HT_1B_. Thus, ω-3 supplementation decreases anxiety of the stressed rats.

Figure 3A and Table 2 show that ω-3 supplementation had an anxiogenic effect on the unstressed rats and significantly reduced corticosterone levels compared to the level in unstressed rats treated with vehicle (Figure 3B). This was unexpected and could be explained by the effects of both ω-3 and serotonin on the HPA axis and neuronal activity in the BNST, respectively. Oral administration of vehicle was a stressor for the rats due because it increased corticosterone levels in the unstressed rats. However, this effect was prevented by ω-3 supplementation (Figure 3B, right side). It is possible that ω-3 prevents stress-induced dendritic hypertrophy in the amygdala of unstressed rats and this decreases corticosterone levels compared to that of unstressed rats treated with vehicle (Figure 3B, right side). Supplementation with ω-3 may increase serotonin levels in the brain of rats [60]. We suggest that to counteract this effect, the expression of the 5-HT_1A_ and 5-HT_1B_ receptors was down-regulated in the BNST of unstressed rats supplemented with ω-3. In this context, neuronal excitability in the BNST may increase because the inhibitory control of serotonin over the BNST is lost. As result, anxiety is enhanced in unstressed rats supplemented with ω-3 (Figure 3A).

Unstressed and stressed rats of control group that were not stimulated had similar corticosterone levels, suggesting that the rats adapted to 21 days of restraint stress (Figure 3B, left side). Previous studies have shown that 3 or 6 hours per day of restraint stress significantly increased corticosterone plasma levels during the first week, while in the second and third weeks of restraint stress the increases of corticosterone levels were less pronounced [61,62]. Therefore, if the effects of 21 days of restraint stress on HPA axis activity and corticosterone levels had been lost, the rats that were subjected to restraint stress and unstressed rats would have had comparable plasma corticosterone levels after exposure to a new uncontrollable stressor (acute swimming). However, control group rats subjected to restraint stress had significantly higher plasma corticosterone levels than unstressed rats following one minute of swimming (Figure 3B). This suggests that one day after the restraint stress ended, unstressed and stressed rats had similar HPA axis activity in an environment without stressors. On the other hand, stressed control group rats still showed higher levels of the HPA axis activity than unstressed rats exposed to a new uncontrollable stressor. This neuroendocrine alteration, which induces maladaptive responses to stressors, is characteristic of stressed animals [42,50].

Corticosterone plasma levels increased for approximately the first seven days of restraint stress [10]. However, the long-term impact of the chronic stress on the neuronal morphology of the lateral amygdala and on anxiety levels were measured after twenty-one days of stress-free recovery [55]. Therefore, chronically stressed rats may have had enhanced anxiety and hyperactivity of the HPA axis at the same time as they were subjected to new stressors like the EPM and swimming in a water maze (Figure 3A,B).

In our experiments, supplementation was applied by oral administration and this method resulted in higher corticosterone levels after acute swimming in unstressed vehicle group rats than those of unstressed control group rats (Figure 3B, right side). This suggests that vehicle treatment, which was applied from weaning to the end of the stress period, was sufficient to induce short-term stress in the supplemented rats. Handing could be comparable to oral administration of vehicle applied before chronic restraint stress protocol. A longer period of handling has gradually less inhibitory effects on the HPA axis activity and significantly decreases the animal’s sensitivity to the restraint stress [63], while a shorter period of handing before applying acute restraint stress results in significantly lower corticosterone and adrenocorticotropic hormone plasma levels than those of rats without handling [63].

The method used to apply the supplementation in our experiments could have induced more profound desensitization of the mechanisms involved in inducing the HPA axis response to restraint stress. This in turn could have resulted in lower corticosterone plasma levels in the stressed rats supplemented with vehicle than in animals that were not supplemented in the control group, after acute swimming (Figure 3B, right side). Desensitization of the HPA axis might involve the loss of CRH receptors in the anterior pituitary, which in turn may induce the corticotrophs to become refractory to CRH hypersecretion during restraint stress.

### The effects of restraint stress and ω-3 fatty acid supplementation on learning

Restraint stress and oral administration of vehicle were stressful for the rats given that the two treatments increased plasma corticosterone levels (Figure 3B). In addition, these treatments impaired learning during the conditioned trials (Figure 4C,D). Lesion studies in the main nuclei of the auditory system that regulates learning, the inferior colliculus (IC, auditory mesencephalon) and the medial geniculate nucleus (MG, auditory thalamus), have demonstrated that the two brain structures are key factors for acquiring aversive memories to auditory cues during fear conditioning in rats [64]. As well, restraint stress induces dendritic atrophy in the IC, MG, and primary auditory cortex, and affects auditory processing [27,65,66]. A recent study using micro Positron Emission Tomography supports these findings, in that chronic mild stress induced a significant decrease in glucose metabolism in the IC, but not in the superior colliculus (visual mesencephalon) [67]. In our study, learning impairment could have been due to stress-induced dendritic atrophy in the IC and/or MG. In support of this idea, unstressed and stressed rats supplemented with vehicle showed significant less learning than animals without supplementation. However, ω-3 supplementation prevented this effect (Figure 4C,D,E), possibly by preventing the stress-induced impairment in the IC and/or MG. In fact, ω-3 fatty acid deficiency impairs active avoidance and decreases the polyunsaturated fatty acid composition in the cellular and subcellular fractions [38]. As well, learning alterations associated with maternal deficiency of α-linolenic acid are prevented by α-linolenic acid supplementation after weaning [68]. Likewise, DHA supplementation prevents learning impairments in rats induced by ω-3 deficiency in rats [30].

Other brain nuclei that are key for acquiring and evoking auditory avoidance conditioned responses are the lateral (LA) and basal amygdala [26]. Therefore, another possible explanation for our results in the 2-AA is that restraint stress and ω-3 supplementation had opposite effects on these nuclei, that restraint stress induced dendritic hypertrophy in the LA and this dendritic change enhanced anxiety-like behaviors by the BNST [54]. On the other hand, ω-3 supplementation may have prevented these morphologic alterations and produced anxiolitic effects in stressed rats. This, in turn could have improved learning, as has been seen with anxiolitic drugs such as midazolam, which facilitates avoidance retrieval in rats [69].

### Possible cellular mechanisms underlying the anti-stress effects of ω-3 fatty acids supplementation

A growing body of evidence suggests that ω-3 PUFA levels in the brain modulate the reactivity and sensitivity to stress [70]. In addition, chronic stress reduces the DHA content in the brain phospholipids and prevents the incorporation of supplemental-DHA in the neuronal membranes [60,71]. We suggest that restraint stress decreases DHA content in the phosoholipid membranes of glutamatergic neurons at the amygdaloid complex, whereas it increases arachidonic acid (AA) content. In support of this idea, studies with monkeys have shown that chronic stress is associated with a higher phosphatidylethanolamine ω-6/ω-3 ratio, suggesting lower ω-3 fatty acid status in stressed animals [72]. AA is released from the phospholipid membranes to the cytoplasm by cytoplasmic phospholipase A2 (cPLA_2_) activity and is transformed into endocannabinoid (eCb), which in turn inhibits GABA release from presynaptic neurons [73-75]. Through this mechanism, chronic stress may increase excitatory neuronal activity in the amygdala and enhance anxiety. On the other hand, ω-3 supplementation may increase DHA content in the phospholipid membranes of excitatory neurons; which in turn decrease AA levels in the cytoplasm of neurons. Thus, inhibitory transmission could be reduced in the amygdala; decreasing anxiety and plasma corticosterone levels in the stressed rats (Figure 3A,B).

The anxiolytic effect of ω-3 supplementation may be related to increased serotonin levels in the brain of chronically stressed rats. Serotonin has a key role in the regulation of anxiety-like behaviors [76]. In the case of our study, ω-3 PUFA supplementation could have enhanced the serotonin level in the brain [76,77].

The positive effect of ω-3 supplementation on the learning could be related to a direct effect of ω-3 on the auditory brain nuclei that modulates fear learning, such as the MG and IC, which are affected in the stressed rats [65,67]. Chronic stress may have decreased proplastic protein levels in the brain nuclei that produce dendritic atrophy. Proplastic proteins are implicated in neurite extension, cell survival and synaptic plasticity [78]. Alternatively, ω-3 supplementation may increase the level of the proteins that prevent dendritic atrophy in the MG and IC of stressed rats. On the other hand, the positive effects of ω-3 fatty acids on learning may have been by a direct effect in the LA, a brain area key for fear learning [26]. Long-term potentiation studies show that auditory fear learning depends on AMPA receptor insertion in the plasmatic membrane of LA neurons [79]. Thus, chronic stress may impair this process in the LA, while ω-3 supplementation could prevent this effect. As well, a mixture of the two mechanisms may be associated with the positive effects of ω-3 fatty acids on the learning of stressed rats.

### Clinical impact of ω-3 fatty acid supplementation on stress-related disorders

Preclinical and clinical studies support the use of ω-3 supplementation in stress-related disorders such as depressive and anxiety disorders. For example, diets rich in ω-3 improve the effects of antidepressants in animal models of depressive-like behaviors [80,81], as well as in patients with major depression [82,83] and anxiety disorders [40]. In this context, we propose that due to its anxiolytic and anti-stress effects, ω-3 supplementation can improve the symptoms of patients with depressive and anxiety disorders. Conversely, diets poor in ω-3 could be a risk factor for developing depressive and anxiety disorders.

## Conclusions

The present findings demonstrate that ω-3 supplementation in an early phase of brain development has strong anti-stress effects, decreasing plasma corticosterone levels and anxiety. In addition, ω-3 supplementation can improve learning in stressed rats. Our results suggest that key brain areas for learning, such as the amygdala and auditory thalamus, could be targets for the positive effects of ω-3 supplementation to improve learning in stressed rats.

## Abbreviations

BNST: Bed nucleus of stria terminalis; CR: Conditioned avoidance response; CRH: Corticotrophin releasing factor; CS: Conditioned stimulus; dB: Decibel; DHA: Docosahexaenoic acid; DRN: dorsal raphé nucleus; EPA: Eicosapentaenoic acid; EPM: Elevated Plus Maze; g: Gram; GR: Glucocorticoid receptor; IC: Inferior colliculus; ITI: Intertrial interval; kHz: Kilohertz; LA: Lateral amygdale; mA: Milliampere; MG: Medial geniculate nucleus; ml: Millilitre; PND: Postnatal days; PUFA: Polyunsaturated fatty acid; sec: Second; μg/dL: Micrograme/Decilitre; US: Unconditioned stimulus; W: Watt; ω-3: Omega-3 fatty acid; ω-6: Omega-6 fatty acid; U: Unstressed; 2-AA: Two-active avoidance conditioning.

## Competing interests

The authors declare that they have no competing interest.

## Authors’ contributions

MAP: Carried out the behavioral, hormonal, and supplementation studies. Participated in the data analyses and drafted the manuscript. GT: Participated in the behavioral studies. ADS: Participated in the data analyses and drafted the manuscript. All authors read and approved the final manuscript
